# Plastin-3 is a diagnostic and prognostic marker for pancreatic adenocarcinoma and distinguishes from diffuse large B-cell lymphoma

**DOI:** 10.1186/s12935-021-02117-1

**Published:** 2021-08-04

**Authors:** Fei Xiong, Guan-Hua Wu, Bing Wang, Yong-Jun Chen

**Affiliations:** Department of Biliary-Pancreatic Surgery, Tongji Hospital, Tongji Medical College, Huazhong University of Science and Technology, Wuhan, 430074 Hubei China

**Keywords:** Pancreatic adenocarcinoma, Bioinformatic analysis, Differentially expressed genes, Biomarker, Diffuse large B-cell lymphoma

## Abstract

**Background:**

Altered Plastin-3 (PLS3; an actin-binding protein) expression was associated with human carcinogenesis, including pancreatic ductal adenocarcinoma (PDA). This study first assessed differentially expressed genes (DEGs) and then bioinformatically and experimentally confirmed PLS3 to be able to predict PDA prognosis and distinguish PDA from diffuse large B-cell lymphoma.

**Methods:**

This study screened multiple online databases and revealed DEGs among PDA, normal pancreas, diffuse large B-cell lymphoma (DLBCL), and normal lymph node tissues and then focused on PLS3. These DEGs were analyzed for Gene Ontology (GO) terms, Kaplan–Meier curves, and the log-rank test to characterize their association with PDA prognosis. The receiver operating characteristic curve (ROC) was plotted, and Spearman’s tests were performed. Differential PLS3 expression in different tissue specimens (n = 30) was evaluated by reverse transcription quantitative polymerase chain reaction (RT-qPCR).

**Results:**

There were a great number of DEGs between PDA and lymph node, between PDA and DLBCL, and between PDA and normal pancreatic tissues. Five DEGs (NET1, KCNK1, MAL2, PLS1, and PLS3) were associated with poor overall survival of PDA patients, but only PLS3 was further verified by the R2 and ICGC datasets. The ROC analysis showed a high PLS3 AUC (area under the curve) value for PDA diagnosis, while PLS3 was able to distinguish PDA from DLBCL. The results of Spearman's analysis showed that PLS3 expression was associated with levels of KRT7, SPP1, and SPARC. Differential PLS3 expression in different tissue specimens was further validated by RT-qPCR.

**Conclusions:**

Altered PLS3 expression was useful in diagnosis and prognosis of PDA as well as to distinguish PDA from DLBCL.

**Supplementary Information:**

The online version contains supplementary material available at 10.1186/s12935-021-02117-1.

## Introduction

Pancreatic ductal adenocarcinoma (PDA) is one of the most malignant and lethal cancers, accounting for 459,000 new cases diagnosed in 2018. The overall 5-year PDA survival rate is approximately 9% [1]. Currently, as the seventh most common cause of cancer death in the USA, PDA is projected to become the second most common cause of cancer death in the USA by 2030 [2]. The development of PDA is associated with many risk factors that are poorly characterized, rendering PDA prevention almost impossible [1]. Moreover, despite recent advancements in our understanding of the tumor’s biology, PDA is still frequently diagnosed at advanced stages of disease (initial diagnosis only occurs in up to 15% of PDA patients with surgically resectable tumors) [1]. Surgical tumor resection is not possible in many cases, and PDA is insensitive to chemoradiotherapy [3, 4]; however, in early stage PDA, preoperative chemotherapy followed by surgical resection is regarded as a curative treatment [5], but it has not been associated with significant improvement in patient outcomes or quality of life in advanced PDA [6]. Thus, there is an urgent need to understand the molecular mechanisms of PDA carcinogenesis and progression in order to develop biomarkers to diagnose PDA early, to predict prognosis and treatment responses, and to design novel strategies for the control of PDA.

In terms of early PDA detection, the differential diagnosis of an abdominal mass, like PDA, is challenging. Primary pancreatic lymphoma (PPL) is an extremely rare form of extranodal malignant lymphoma, accounting for less than 0.5% of pancreatic neoplasms and 1% of extranodal lymphomas [7]. The most common histological type of PPL is diffuse large B cell lymphoma (DLBCL), which accounts for nearly 60% of all PPL cases [8]. PPL manifests as an abdominal mass that is similar to PDA [9]; however, chemotherapy (like the CHOP regimen) is the preferred treatment option for PPL patients, so the differential diagnosis is crucial. However, if PPL was misdiagnosed, the patients would undergo unnecessary surgery, which is not an ideal treatment option for the patients [10–12].

Plastin-3 (PLS3), belonging to a family of actin-binding proteins, functions to inhibit cofilin-mediated depolymerization of the actin fiber, which may have an important role in the epithelial–mesenchymal transition (EMT) [13]. Altered PLS3 expression was reported to be associated with human carcinogenesis, including PDA, and the detection of PLS3 expression may predict prognosis in various human cancers [13–19]. Molecularly, PLS3 regulates the PI3K/AKT signaling pathway and the EMT in tumor cells [13, 14].

In this study, we performed bioinformatical analyses of multiple datasets to assess differentially expressed genes (DEGs) in PDA, normal pancreas, DLBCL, and normal lymph node tissue. We then focused on PLS3 as a biomarker for the early diagnosis of PDA, as well as the prediction of prognosis and differential diagnosis from DLBCL. We expect to provide useful information regarding PLS3 as a biomarker for PDA, in future validation studies.

## Materials and methods

### Searching and downloading of microarray data

**Fig. 1:**
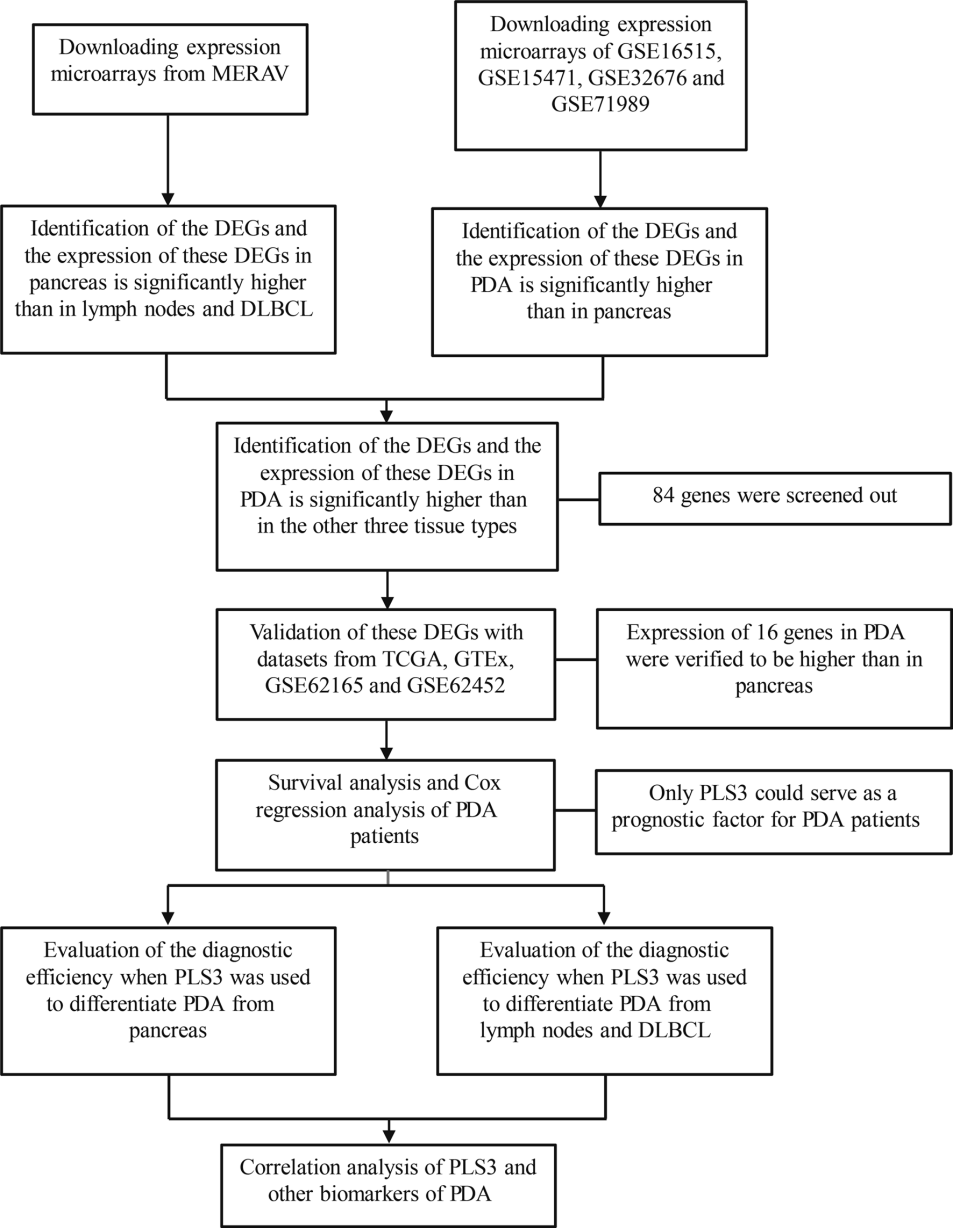
Illustration of our search criteria and flow diagram

In this study, we searched and downloaded multiple data from various databases [i.e., the Gene Expression Omnibus (GEO; https://www.ncbi.nlm.nih.gov/geo), the Metabolic gEne RApid Visualizer (MERAV, http://merav.wi.mit.edu), The Cancer Genome Atlas (TCGA; https://portal.gdc.cancer.gov/), and the UCSC Xena (https://xena.ucsc.edu/)] using our search criteria (Fig. 1). The GEO datasets included GSE16515, GSE15471, GSE32676, GSE71989, GSE71729, GSE62165, and GSE62452, while GSE16515, GSE2109, and GSE7307 were used for batch-normalization with the Metabolic Gene Rapid Visualizer (MERAV; http://merav.wi.mit.edu/). The microarray data on PDA and DLBCL cell lines were downloaded from the MERAV [20], and microarray data from TCGA and the Genotype-Tissue Expression (GTEx) were obtained from the UCSC Xena database (https://xenabrowser.net/). Each dataset contained various numbers of PDA, normal pancreas, lymph node, and DLBCL tissue samples (Tables 1 and 2).

**Table 1:** The GEO datasets used in this study

Accession number	Platform	Pancreas	PAAD	Lymph nodes
GSE15471	GPL570	39	39	0
GSE16515	GPL570	16	36	0
GSE32676	GPL570	7	25	0
GSE71989	GPL570	8	13	0
GSE71729	GPL20769	46	145	10
GSE62165	GPL13667	13	118	0
GSE62452	GPL6244	61	69	0

**Table 2:** The MERAV data

Tissue	Source	Platform	Number	Sample ID
Pancreas	GSE16515	GPL570	15	GSM414928, GSM414930, GSM414934, GSM414938, GSM414940, GSM414942, GSM414947, GSM414953, GSM414955, GSM414957, GSM414963, GSM414966, GSM414970, GSM414972, GSM414975
	GSE7307	GPL570	1	GSM175950
Lymph nodes	GSE7307	GPL570	4	GSM176431, GSM176432, GSM176432, GSM176434
DLBCL	GSE2109	GPL570	4	GSM53026, GSM102466, GSM152566, GSM179927

### Identifications of DEGs

To identify the DEGs, we processed the downloaded database data with the “limma” R package using the R version 3.6.3 (https://www.r-project.org) according to a previous study [21]. The false-positive results were corrected by adjusting the P-values (adj. P) during Benjamini–Hochberg analysis. The fold-change value was obtained from the logarithm (logFC) analysis. The threshold for each DEG was set as an adj. P < 0.05 and |logFC| > 1. The R package “sva” was used to adjust batch effects among the GSE16515, GSE15471, GSE32676, and GSE71989 datasets [22]. DEGs were visualized with the “pheatmap” R package. An online Venn diagram (http://bioinformatics.psb.ugent.be/webtools/Venn/) tool was used to identify intersections among gene sets.

### Gene ontology (GO) analysis

GO analysis was applied to define genes and their products (mRNA or proteins), to identify unique biological properties of high-throughput transcriptome or genome data. These analyses were conducted with the “clusterProfiler” R package. With the cut‑off criterion for a significant function was set as an adj. P < 0.05 [23], the GO terms were classified into three groups: biological processes (BP), cellular components (CC), and molecular functions (MF). Data were plotted with the “GOplot” R package [24].

### Tissue samples, RNA isolation, and reverse transcription quantitative polymerase chain reaction (RT-qPCR)

Ten pairs of PDA and normal pancreas samples and five pairs of DLBCL and normal lymph node samples were obtained from Tongji Hospital of Huazhong University of Science and Technology, Wuhan, China. This study was approved by the Ethics Committee of Tongji Hospital (detailed in Additional file 1: Table S1). Tissue samples were subjected to total RNA isolation using the RNA isolater Total RNA Extraction Reagent (Vazyme, Nanjing, China) and reverse-transcribed into cDNA using the HiScript III RT SuperMix for qPCR (+gDNA wiper) (Vazyme) according to the manufacturer’s instructions. qPCR was then performed using the ChamQ Universal SYBR qPCR Master Mix (Vazyme) in the iQ5™ quantitative PCR detection system (Bio-Rad, Richmond, CA, USA). The primers were: PLS3, 5′-AAGACCTTCCGCAAAGCAATC-3′ and 5′-TGTTCCTTCGCTGGACAACTC-3′, and ACTB, 5′-GTCCACCGCAAATGCTTCTA-3′ and 5′-TGCTGTCACCTTCACCGTTC-3′. The qPCR data were quantified using the 2^−∆∆Ct^ method.

### Statistical analyses

To assess bivariate correlations between variables, we determined Spearman’s rank correlation coefficient (r_s_) using the R 3.6.3 package and SPSS 21.0 (SPSS Inc., Chicago, IL, USA). The output results were visualized using the “corrplot” R package, and P < 0.05 was considered as statistically significant. Kaplan–Meier curves were plotted for 176 PDA patients, the data of which were obtained from TCGA and downloaded from the UCSC Xena site (https://xena.ucsc.edu), then analyzed with the log-rank test to calculate overall survival for groups of patients after stratification for DEGs. Another dataset was downloaded from the complementary data available on the R2: Genomics Analysis and Visualization Platform (http://r2.amc.nl) and International Cancer Genome Consortium (ICGC; https://dcc.icgc.org/) [25]. The data were analyzed by using for Kaplan–Meier analysis with the “survival” R package. For data analyses, all patients were divided into high vs. low groups, depending on the median expression level of each DEG (cut‑off P < 0.05), using the log-rank test. We also performed univariate and multivariate Cox regression analyses using the “survival” R package. The association between PLS3 expression and clinicopathological features was analyzed with a Chi-square test (P < 0.05). To predict the utility of PLS3 in diagnosing PDA, we plotted the receiver operating characteristic curve (ROC) with the “pROC” R package [26], then calculated the area under the curve (AUC) with SPSS 21.0.

## Results

### Identification of PDA-related DEGs using various online datasets

**Fig. 2:**
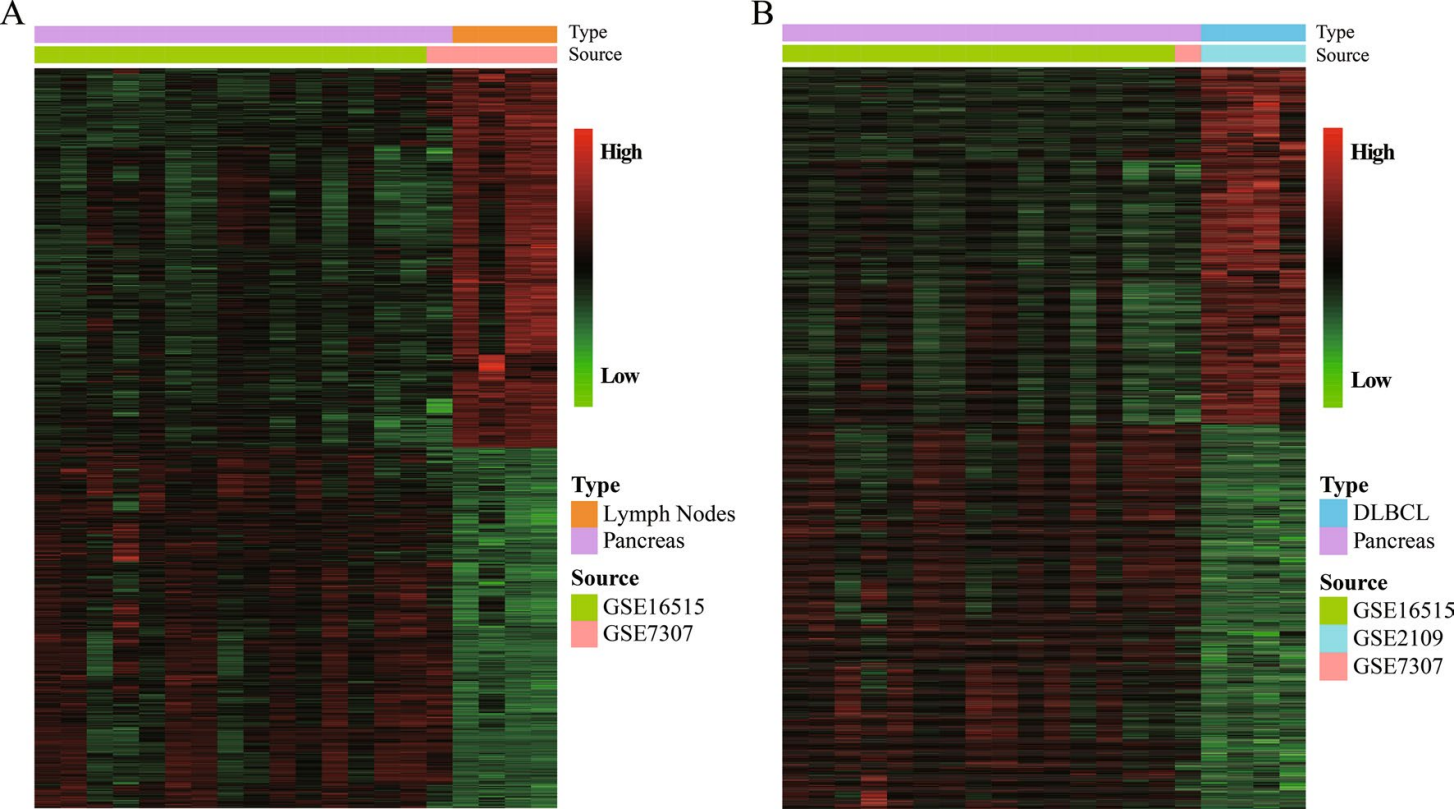
The hierarchical cluster heatmaps for DEGs in the MERAV datasets. **A** DEGs between normal pancreas and lymph node tissue. **B** DEGs between normal pancreas and DLBCL tissue. The gradual change from red to green represents changes in gene expression from high to low. The black color refers to no difference in gene expression. *DEG* differentially expressed gene, *MERAV* the Metabolic Gene Rapid Visualizer, *DLBCL* diffuse large B-cell lymphoma

**Fig. 3:**
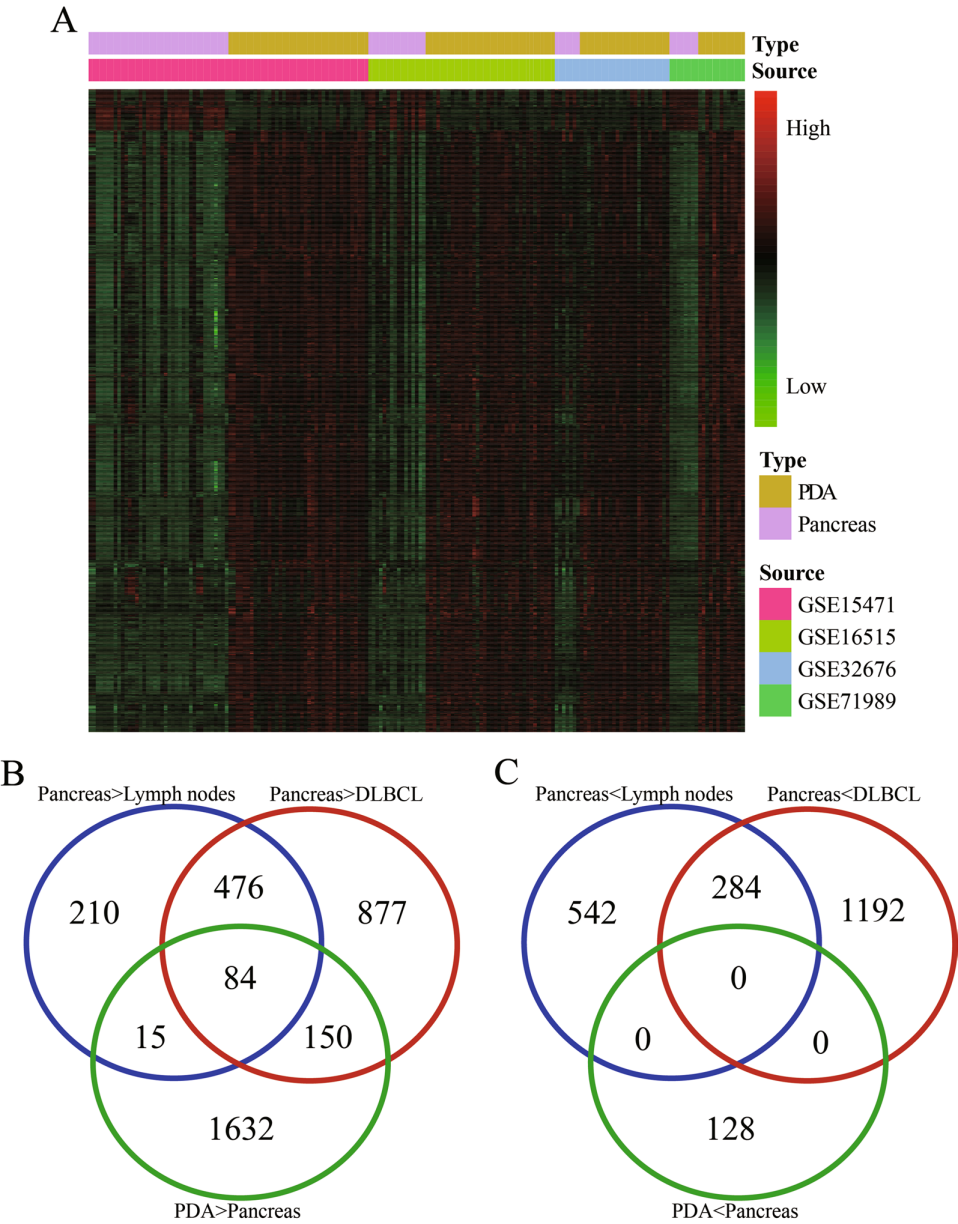
DEGs identified from the batched GEO datasets with the Venn diagrams. **A** The hierarchical cluster heatmaps of DEGs between PDA and normal pancreas from the four GEO batched datasets. The gradual change from red to green represents changes in gene expression from high to low. The black color indicates no difference in gene expression. **B** The intersection among “Pancreas > Lymph Nodes”, “Pancreas > DLBCL” and “PDA > Pancreas” groups. **C** The intersection among “Pancreas < Lymph Nodes”, “Pancreas < DLBCL” and “PDA < Pancreas” groups. *DEG* differentially expressed gene, *GEO* Gene Expression Omnibus, *PDA* pancreatic ductal adenocarcinoma, *DLBCL* diffuse large B-cell lymphoma

In this study, we downloaded multiple datasets and performed bioinformatic analyses to identify DEGs in PDA, normal pancreas, DLBCL, and normal lymph node tissue. With use of the MERAV dataset, we found a total of 1611 DEGs between pancreatic and lymph node samples and 3063 DEGs between pancreatic and DLBCL samples (Fig. 2A, B and Table 3). Using 113 PDA and 70 pancreas samples, we compared the DEGs identified with the MERAV dataset with those identified with the GEO dataset (GSE16515, GSE15471, GSE32676, and GSE71989) (Table 1). This approach ultimately resulted in the identification of 1881 upregulated DEGs and 128 downregulated DEGs (Table 3 and Fig. 3A).

**Table 3:** DEGs among PDA, normal pancreas, lymph node, and DLBCL tissues

	Differences in expression between samples	Number of DEGs	Total number
Pancreas vs. lymph nodes	Pancreas > lymph nodes	785	1611
	Pancreas < lymph nodes	826	
Pancreas vs. DLBCL	Pancreas > DLBCL	1476	3063
	Pancreas < DLBCL	1587	
PDA vs. pancreas	PDA > pancreas	1881	2009
	PDA < pancreas	128	

*DEG* differentially expressed gene, *DLBCL* diffuse large B-cell lymphoma, *PDA* pancreatic ductal adenocarcinoma

We created a search filter for these DEGs in PDA from normal pancreas, lymph node, and DLBCL using the intersection calculation and the transitivity of inequality relation of gene expression sourcing from GEO and MERAV for the “Pancreas > Lymph Nodes”, “Pancreas > DLBCL”, and “PDA > Pancreas” groups. The intersection showed that 84 DEGs were significantly higher in PDA than in the other three tissue types (Fig. 3B); however, we did not identify any DEGs among the other three groups (Fig. 3C).

### Functional GO term analysis of these DEGs

**Fig. 4:**
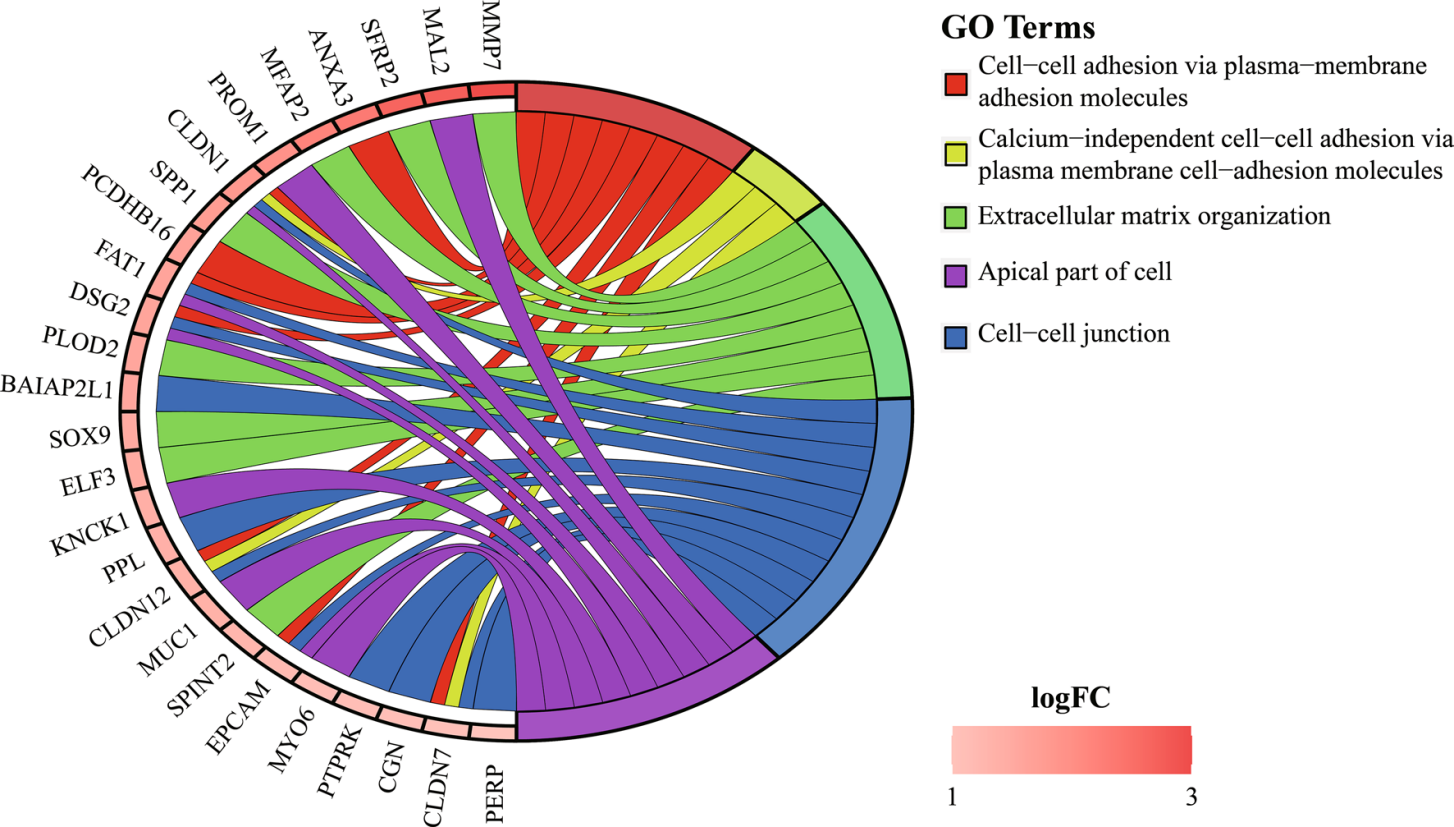
Gene Ontology terms of the 84 DEGs identified with interaction analysis. Changes in red color saturation represent differences in logFC values

We focused solely on these 84 DEGs for the GO term analysis and found that the top six terms (“cell–cell junction”, “cell adhesion molecule binding”, “apical part of cell”, “lateral plasma membrane”, and “desmosome”) were significantly associated with PDA development (Fig. 4 and Table 4).

**Table 4:** The remarkably enriched GO terms for these DEGs

Ontology	ID	Description	Count^a^	adj. P	Gene ID
CC	GO:0005911	Cell–cell junction	12	6.95E−05	PERP/PPL/EPCAM/FAT1/CLDN1/BAIAP2L1/DSG2/CLDN7/CLDN12/PTPRK/CGN/PDLIM3
MF	GO:0050839	Cell adhesion molecule binding	11	0.002369034	SFRP2/PPL/SPP1/EPCAM/OLFM4/BAIAP2L1/TSPAN8/DSG2/CCN2/PROM1/CGN
CC	GO:0045177	Apical part of cell	9	0.002472218	EPCAM/FAT1/CLDN1/MYO6/MUC1/DSG2/KCNK1/PROM1/MAL2
CC	GO:0016324	Apical plasma membrane	8	0.002817074	EPCAM/FAT1/CLDN1/MUC1/DSG2/KCNK1/PROM1/MAL2
CC	GO:0016328	Lateral plasma membrane	4	0.003551437	EPCAM/CLDN1/DSG2/CLDN12
CC	GO:0030057	Desmosome	3	0.004170446	PERP/PPL/DSG2
CC	GO:0005923	Bicellular tight junction	5	0.004170446	EPCAM/CLDN1/CLDN7/CLDN12/CGN
CC	GO:0070160	Tight junction	5	0.004305753	EPCAM/CLDN1/CLDN7/CLDN12/CGN
BP	GO:0030198	Extracellular matrix organization	10	0.006175832	MMP7/SFRP2/SOX9/SPP1/PLOD2/MFAP2/ELF3/CCN2/SPINT2/CCDC80
CC	GO:0043296	Apical junction complex	5	0.006294613	EPCAM/CLDN1/CLDN7/CLDN12/CGN
BP	GO:0043062	Extracellular structure organization	10	0.010219773	MMP7/SFRP2/SOX9/SPP1/PLOD2/MFAP2/ELF3/CCN2/SPINT2/CCDC80
BP	GO:0098742	Cell–cell adhesion via plasma-membrane adhesion molecules	8	0.011695026	ANXA3/EPCAM/FAT1/CLDN1/DSG2/CLDN7/CLDN12/PCDHB16
CC	GO:0005884	Actin filament	4	0.020148378	PLS3/MYO6/PLS1/PDLIM3
MF	GO:0001968	Fibronectin binding	3	0.023328786	SFRP2/CCN2/CCDC80
CC	GO:0062023	Collagen-containing extracellular matrix	7	0.023568462	SFRP2/LGALS4/ASPN/MFAP2/CCN2/F3/CCDC80
MF	GO:0003779	Actin binding	8	0.042702752	PLS3/BAIAP2L1/MYO6/PLS1/ENC1/CGN/MLPH/PDLIM3
MF	GO:0002162	Dystroglycan binding	2	0.044071511	AGR2/AGR3
BP	GO:0016338	Calcium-independent cell–cell adhesion via plasma membrane cell-adhesion molecules	3	0.047603714	CLDN1/CLDN7/CLDN12

*BP* biological processes, *CC* cellular component, *MF* molecular functions

^a^The “Count” refers to the numbers of DEGs that were enriched in the corresponding functional category

### Validation of these DEGs using TCGA and GTEx data

**Fig. 5:**
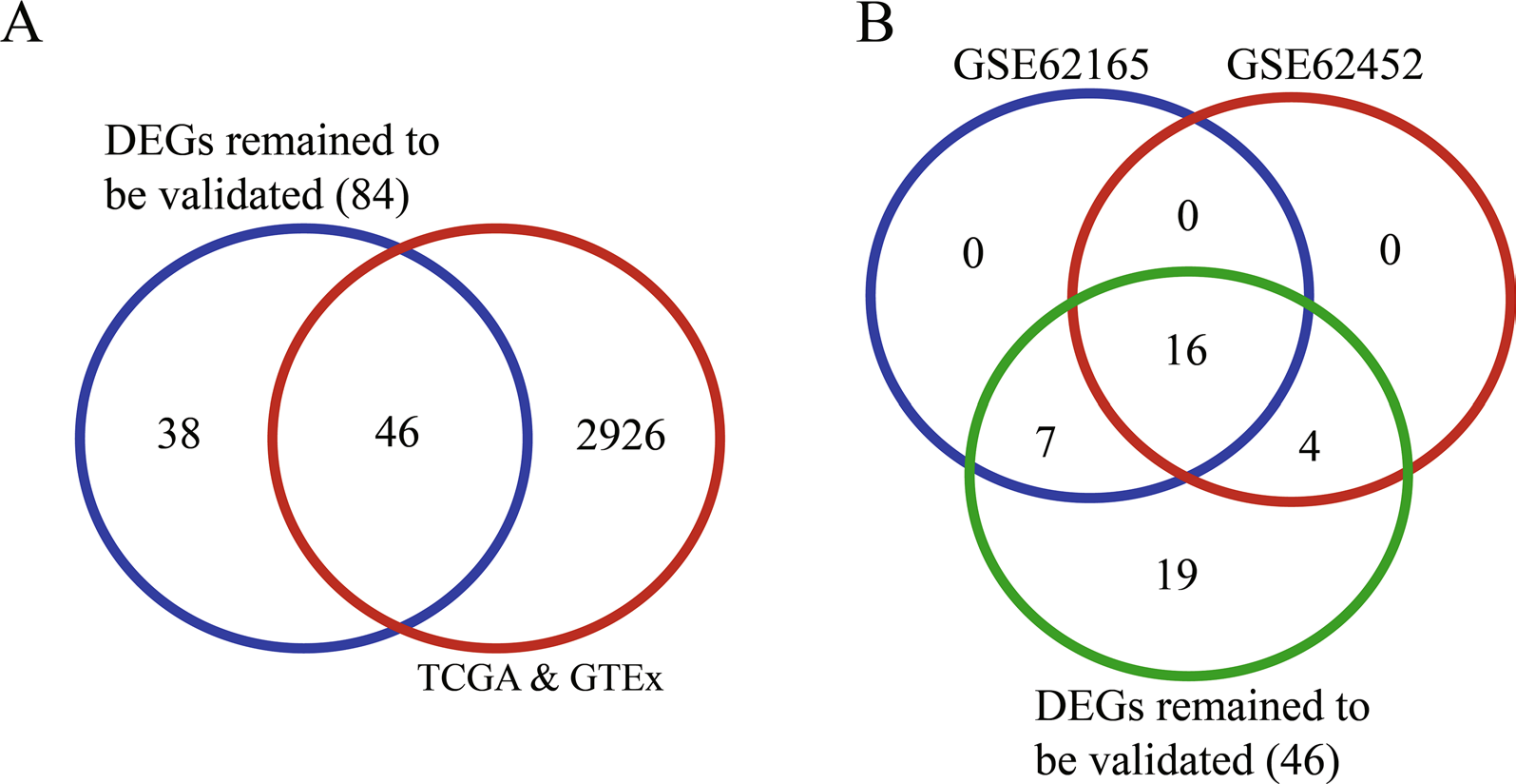
Validation of DEGs between PDA and normal pancreatic tissue among TCGA, GTEx, and GEO datasets. **A** The intersection between the 84 DEGs in the GEO datasets and DEGs from the TCGA and GTEx datasets. Forty-six DEGs were identified after comparison of all three databases. **B** Validation of these 46 DEGs with GSE62165 and GSE62452. Sixteen out of 46 DEGs were confirmed. *DEG* differentially expressed gene, *PDA* pancreatic ductal adenocarcinoma, *TCGA* The Cancer Genome Atlas, *GTEx* Genotype-Tissue Expression, *GEO* Gene Expression Omnibus

To validate the DEGs from the MERAV and GEO datasets, we searched and downloaded mRNA sequencing data on 178 PDA and 171 normal pancreas samples from TCGA and the GTEx database. Among a total of 2971 upregulated DEGs in the MERAV and GEO datasets, 46 were confirmed as highly expressed in PDA in TCGA and GTEx datasets. These DEGs may serve as indicators for differentiation between PDA and the other three tissue types (Fig. 5A). An intersectional analysis of GSE62165 (13 pancreas and 118 PDA samples) and GSE62452 (61 pancreas and 69 PDA samples) revealed that 16 of these 46 DEGs were significantly overexpressed in PDA (Fig. 5B and Table 1).

### Association of DEGs with PDA prognosis

**Fig. 6:**
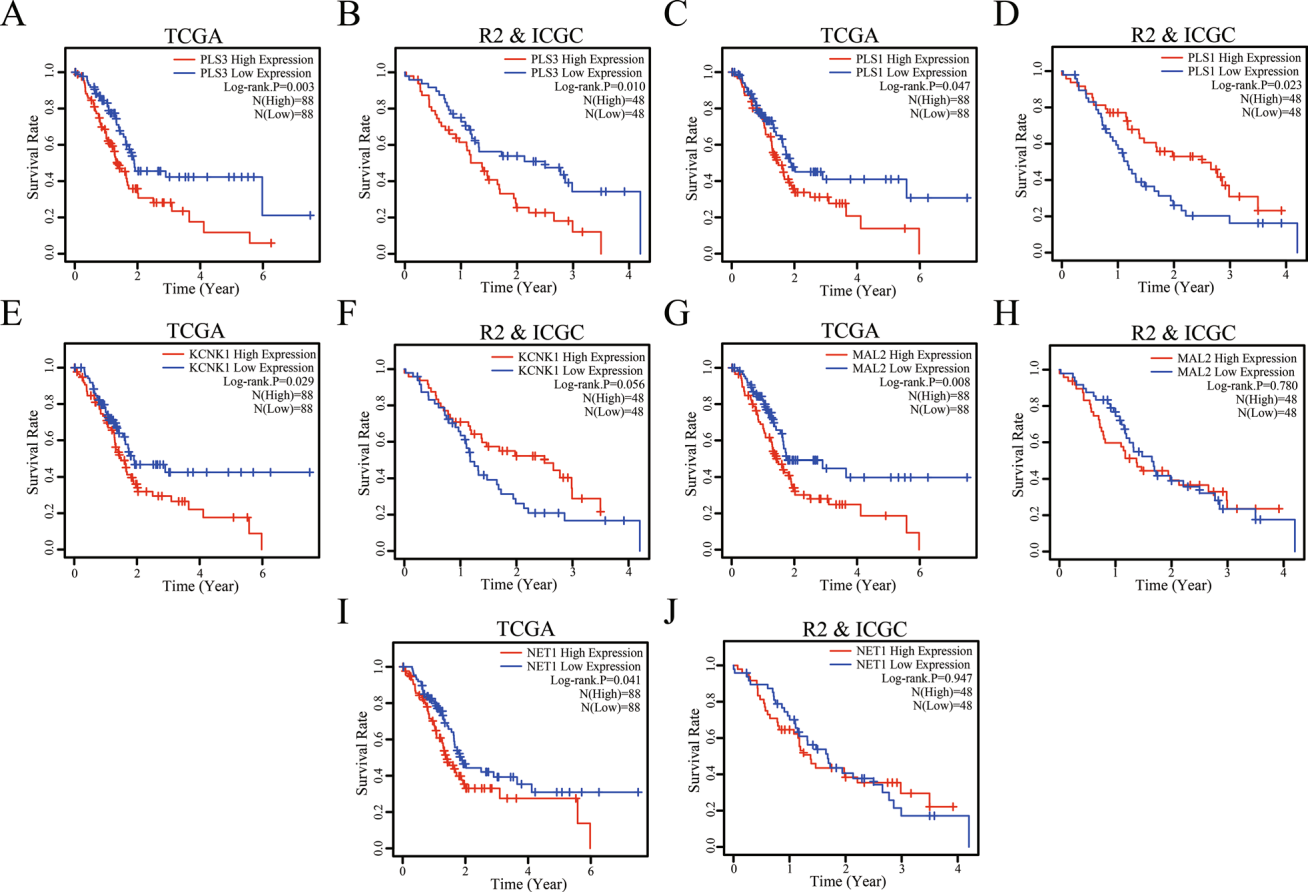
Association of NET1, KCNK1, MAL2, PLS1, and PLS3 expression with PDA prognosis, as determined with TCGA, R2, and ICGC data. **A**, **C**, **E**, **G**, and **I** Kaplan–Meier curves and log-rank test results for PDA patients from TCGA. **B**, **D**, **F**, **H**, and **J** Kaplan–Meier curves and log-rank test results for PDA patients from the R2 and ICGC. Both studies show that patients with high PLS3-expressing PDA had poorer overall survival. *PDA* pancreatic ductal adenocarcinoma, *TCGA* The Cancer Genome Atlas, *R2* The R2 Genomics Analysis and Visualization Platform, *ICGC* International Cancer Genome Consortium

We investigated the association of these 16 DEGs with PDA prognosis by plotting the Kaplan–Meier curves and performing the log-rank test on data for the 176 PDA patients obtained from TCGA (Additional file 1: Table S1). Our data showed that five (NET1, KCNK1, MAL2, PLS1, and PLS3) of these 16 DEGs were associated with poor overall survival (OS) in PDA patients (Fig. 6A, C, E, G, and I). However, only PLS3 data were verified by the survival data integrated from the R2 and ICGC databases (Additional file 1: Table S1) . We also had contradictory data on PLS1, i.e., data from TCGA showed that PLS1 expression was associated with poor survival, but data from another dataset showed the opposite result, that a decrease in PLS1 expression was associated with poor patient survival (Fig. 6B, D, F, H, J). In this regard, we removed the PLS1 data from our subsequent data analyses. The tumor N classification was reversely associated with survival of PDA patients (Additional file 2: Figure S1), but we didn’t find a correlation between PLS3 expression and the clinicopathological features of PDA (Table 5). The results of univariate and multivariate Cox analyses showed that PDA N classification (P = 0.004) and PLS3 expression (P = 0.037) were significant risk factors in developing PDA (Additional file 1: Table S1). The results of multivariate analysis showed that PDA N classification (P = 0.036) and PLS3 expression (P = 0.026) were independent predictors of PDA survival (Table 6).

**Table 5:** Association of PLS3 expression with clinicopathological features in PDA patients from TCGA

Variables	Cases (number, %)	PLS3 level (number, %)		*P*
		High expression N = 83	Low expression N = 84	
Age (years)				
≤ 55	33 (19.8)	20 (60.6)	13 (39.4%)	0.16
> 55	134 (80.2)	63 (47.0)	71 (53.0)	
Histologic grade				
G1/G2	117 (70.1)	57 (48.7)	60 (51.3)	0.69
G3/G4	50 (29.9)	26 (52.0)	24 (48.0)	
N classification				
N0	47 (28.1)	20 (42.6)	27 (57.4)	0.24
N1	120 (71.9)	63 (52.5)	57 (47.5)	
T classification				
T1/T2	28 (16.8)	12 (42.9)	16 (57.1)	0.42
T3/T4	139 (83.2)	71 (51.1)	68 (48.9)	
Gender				
Female	76 (45.5)	37 (48.7)	39 (51.3)	0.81
Male	91 (54.5)	46 (50.5)	45 (49.5)	
Stage				
Stage I/II	160 (95.8)	79 (49.4)	81 (50.6)	0.68
Stage III/IV	7 (4.2)	4 (57.1)	3 (42.9)

**Table 6:** Univariate and multivariate Cox regression analyses of clinicopathological features associated with survival in PDA patients from TCGA

Parameter	Univariate analysis			Multivariate analysis		
	HR	HR 95% CI	P	HR	HR 95% CI	P
Age	1.403	0.814–2.416	0.121			
Histologic grade	1.424	0.919–2.205	0.113			
N classification	2.180	1.283–3.706	*0.004*	1.864	1.042–3.332	*0.036*
T classification	1.838	0.948–3.562	0.071			
Gender	0.781	0.514–1.187	0.247			
Stage	0.802	0.253–2.546	0.708			
PLS3 expression	1.566	1.028–2.385	*0.037*	1.635	1.059–2.524	*0.026*

*HR* hazard ratio, *CI* confidence interval

Italicized value indicates P < 0.05

### Accuracy of PLS3 expression in the diagnosis of PDA

**Fig. 7:**
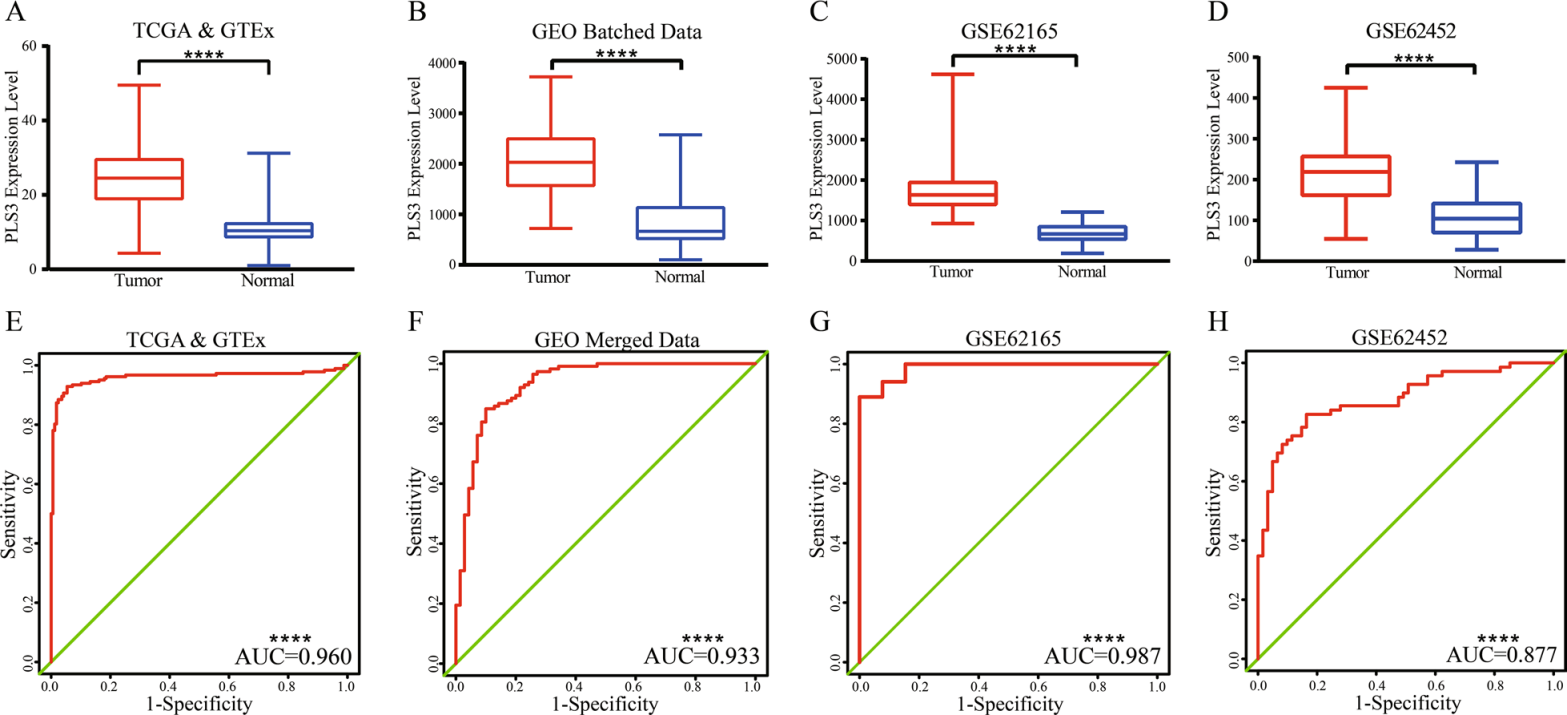
Diagnostic values of PLS3 in PDA vs. normal pancreas. **A**–**D** PLS3 expression in PDA. PLS3 levels were higher than those of normal pancreas in all four datasets. **E**–**H** ROC curves for PLS3 expression in the four datasets. *PDA* pancreatic ductal adenocarcinoma, *ROC* receiver operating characteristic curve. ****P < 0.0001 by Student’s *t*-test

To assess the diagnostic value of PLS3 expression for PDA, we plotted ROC curves and found that PLS3 expression was significantly elevated in PDA (Fig. 7A–D). The diagnostic efficiency of PLS3 in distinguishing PDA from normal pancreas was moderate, with AUC 0.7–0.9 in GSE62452 and high (0.9–1.0) in the other three datasets (Fig. 7E–H).

**Fig. 8:**
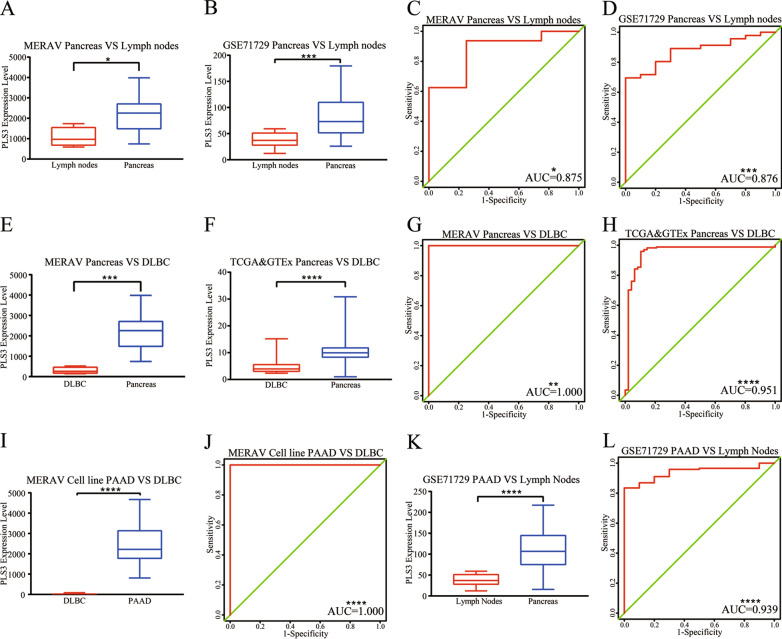
Diagnostic value of PLS3 in PDA and pancreas vs. lymph nodes and DLBCL. **A**, **B** Level of PLS3. PLS3 was significantly higher in the normal pancreas than in the lymph nodes in the MERAV and GSE71729 datasets. **C**, **D** ROC curves of PLS3 in the MERAV and GSE71729 datasets. **E**, **F** Level of PLS3. PLS3 was significantly higher in normal pancreas than in DLBCL in the MERAV, TCGA, and GTEx datasets. **G**, **H** ROC curves for PLS3 in the MERAV, TCGA, and GTEx datasets. **I** Level of PLS3. PLS3 expression was notably higher in PDA than in DLBCL in the cell-line data from the MERAV. **J** ROC curve of PLS3 in the cell line data. **K** PLS3 level in GSE71729. PLS3 expression was higher in PDA than in lymph nodes. **L** ROC curve of PLS3 in GSE71729. *PDA* pancreatic ductal adenocarcinoma, *MERAV* Metabolic Gene Rapid Visualizer, *ROC* receiver operating characteristic curve, *DLBCL* diffuse large B-cell lymphoma, *TCGA* The Cancer Genome Atlas, *GTEx* Genotype-Tissue Expression; ****P < 0.0001, ***P < 0.001, **P < 0.01, and *P < 0.05, analyzed by Student’s *t*-test

We found that PLS3 expression was higher in pancreas than in lymph nodes in the MERAV data (Fig. 8A). The diagnostic efficiency of PLS3 was moderate (Fig. 8B). The examination of 46 normal pancreas and 10 normal lymph node samples revealed a similar result for GSE71729 (Fig. 8C, D and Table 1).

The diagnostic value of PLS3 expression was used to differentiate pancreas from DLBCL in 171 pancreas samples from the TCGA and GTEx, 16 pancreas samples from the MERAV, 48 DLBCL samples from the TCGA, and 4 DLBCL samples from the MERAV. Our data showed that PLS3 was overexpressed in normal pancreas and that the diagnostic efficiency of PLS3 was high (Fig. 8E–H). To verify higher PLS3 expression in PDA than in DLBCL and lymph nodes, we analyzed expression data from GSE71729 (145 PDA and 10 lymph node samples) and cell line data from the MERAV (58 PDA and 17 DLBCL samples). The results revealed that PLS3 expression in PDA was dramatically higher than in DLBCL and normal pancreatic tissues (Fig. 5B, 8I, K) and that the diagnostic efficiency of PLS3 was high (Fig. 8J, L). These data indicate that PLS3 could serve as an effective diagnostic marker to differentiate PDA not only from normal pancreas but also from DLBCL and lymph nodes.

### Association of PLS3 with known prognostic and diagnostic markers in PDA

**Fig. 9:**
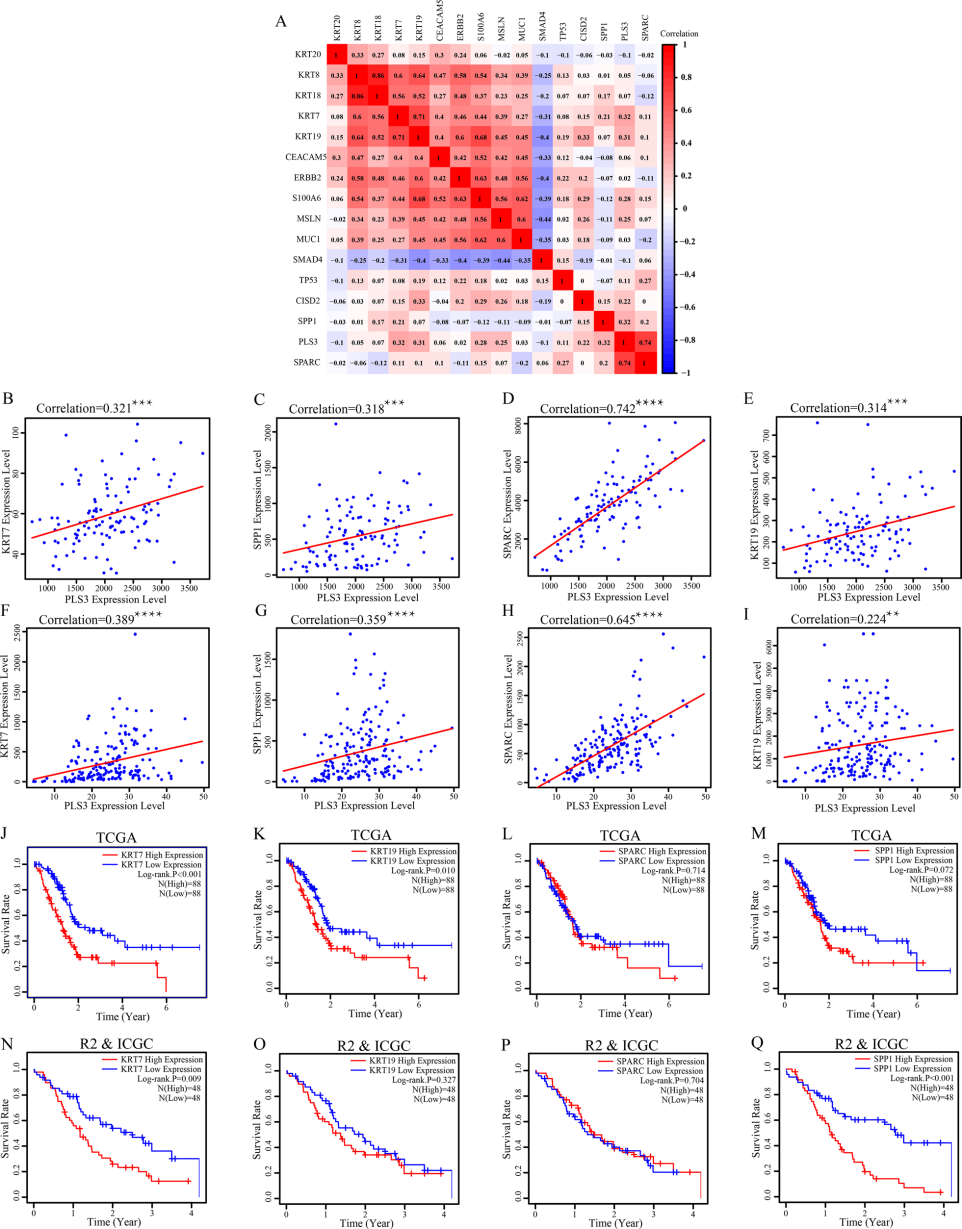
Correlation of PLS3 with other PDA markers. **A** Correlation heatmap reporting Spearman correlation values for each comparison. The bar on the left represents the color legend of Spearman correlation values calculated for each paired PDA marker and PLS3 value. **B**–**E** The correlation of PLS3 with KRT7, KRT19, SPP1, and SPARC in the batched GEO dataset. **F**, **I** The correlation of PLS3 with KRT7, KRT19, SPP1, and SPARC in TCGA. **J**–**M** The Kaplan–Meier curves and the log-rank test results of KRT7, KRT19, SPP1 and SPARC for PDA patients from TCGA. **N**–**Q** Kaplan–Meier curves and log-rank test results of KRT7, KRT19, SPP1 and SPARC for PDA patients from the R2 and ICGC. *PDA* pancreatic ductal adenocarcinoma, *GEO* Gene Expression Omnibus, *TCGA* The Cancer Genome Atlas, *R2* The R2 Genomics Analysis and Visualization Platform, *ICGC* International Cancer Genome Consortium. ****P < 0.0001 and *** P < 0.001 by Student’s *t*-test

To further elucidate the role of PLS3 expression in PDA, we selected various biomarkers that were previously used to diagnosis PDA or to predict prognosis in affected patients [27–29] and calculated the r_s_ values. Using the batched dataset from GEO data, we found that KRT7 (also known as CK7) and SPP1 (secreted phosphoprotein 1) were associated with PLS3 expression (Fig. 9A), while SPARC (secreted protein acidic and rich in cysteine) had a strong association with PLS3 expression in PDA (Fig. 9B–D). These patterns were also confirmed in TCGA PDA data (Fig. 9F–H). TCGA data revealed a weak association between KRT19 (another PDA marker, also known as CK19) with PLS3, while the GEO dataset showed a moderate association (Fig. 9E, I). We also performed Kaplan–Meier analysis. KRT7 was confirmed to be associated with prognosis in PDA patients in both datasets (Fig. 9J, N). KRT19 and SPP1 were validated by only one dataset (Fig. 9K, L, O, P). There was insufficient evidence to confirm the association between SPARC expression and OS in PDA patients (Fig. 9M, Q).

### Validation of differential PLS3 expression in different tissue specimens

**Fig. 10:**
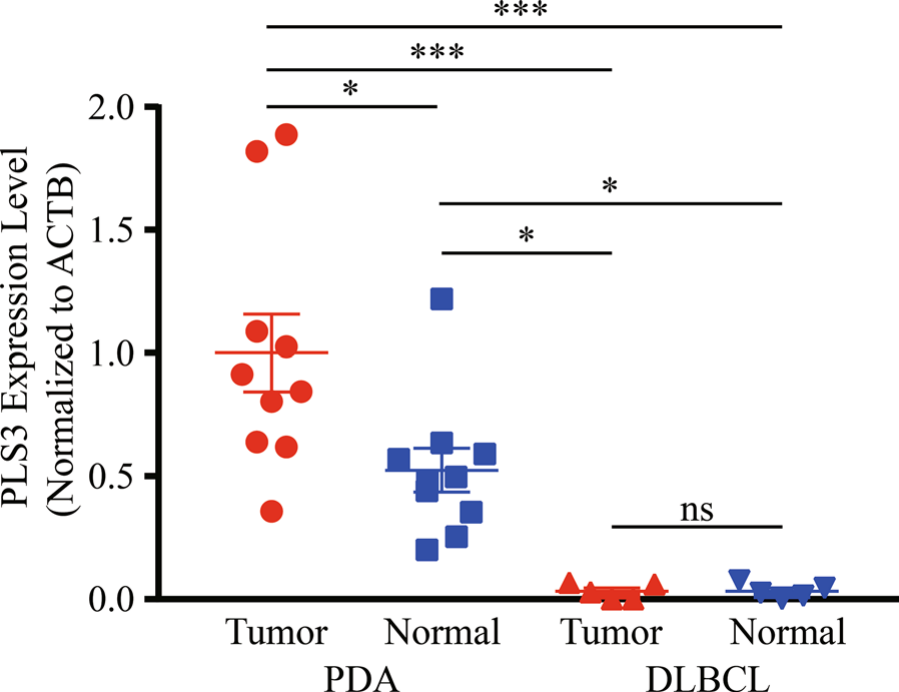
RT-qPCR validation of differential PLS3 expression in tissue specimens. Ten pairs of PDA and normal pancreas and five pairs of DLBCL and normal lymph node samples were subjected to total RNA isolation, reverse-transcribed into cDNA, and then subjected to qPCR. Data were quantified using the 2^−∆∆Ct^ method. ***P < 0.001 and *P < 0.05 by ANOVA

We performed RT-qPCR analysis of PLS3 in ten PDA samples, ten normal pancreas samples, five DLBCL samples, and five lymph node samples (Additional file 1: Table S1). We found that the level of PLS3 mRNA in PDA was significantly higher than that in normal pancreas, DLBCL, and lymph node samples. PLS3 expression in normal pancreas was higher than that in DLBCL and lymph node samples. Intriguingly, PLS3 expression was similar in DLBCL and lymph nodes (Fig. 10).

## Discussion

In the current study, we bioinformatically analyzed the DEGs among PDA, normal pancreas, DLBCL, and normal lymph node tissue samples by downloading the corresponding data from multiple online databases. We narrowed our analysis for association with PDA prognosis to those DEGs that occurred in all datasets, then focused on PLS3 as a biomarker for the early diagnosis of PDA, the prediction of prognosis, and differentiation from DLBCL. Our current study identified a great number of DEGs among these tissue samples, and our intersectional analysis narrowed them down to 84 DEGs, expression of which was significantly higher in PDA than in the three other types of tissue samples. These 84 upregulated DEGs were related to six different GO terms (the cell–cell junction, cell adhesion molecule binding, apical part of the cell, lateral plasma membrane, and desmosome). Moreover, cross-analysis of the TCGA data on 178 PDA and 171 normal pancreas samples verified 46 upregulated DEGs, 16 of which were significantly overexpressed in PDA; however, only five of these 16 DGEs showed significant association with poor PDA prognosis, and only PLS3 was further confirmed on R2 and ICGC database analysis. Our multivariate analysis confirmed that PDA N classification and PLS3 expression were independently predictors for OS among patients with PDA. PLS3 may also be used to diagnose PDA. In conclusion, our current data demonstrate that the detection of PLS3 expression may be used effectively to diagnose PDA, to predict OS in PDA patients, and to distinguish PDA from DLBCL. However, future study is warranted to verify our current data on the use of PLS3 as a biomarker for PDA.

PDA is one of the most malignant and lethal malignancies, and early diagnosis is crucial in controlling this deadly cancer. Surgical resection is the only method proven to control PDA clinically. Nevertheless, DLBCL is usually treated with chemotherapy in the clinic. It is very important to make a differential diagnosis between PDA and pancreatic DLBCL because they have similar clinical appearance and medical imaging [11, 12, 20]. Due to the anatomic location of the pancreas, it is difficult to access and has a risk of intraoperative injury, while biopsy via surgery or laparoscopic surgery might not be the best choice. The EUS-FNA (endoscopic ultrasound-guided fine-needle aspiration biopsy) could be used to take a biopsy of peripancreatic masses, but as an invasive manipulation, the patients still have to take a risk of postoperative complications. Besides, a technical obstacle is an unavoidable issue [30]. By contrast, despite lower accuracy, serum tumor marker is non-invasive and easier to perform. To date, there have been numerous studies investigating biomarkers for PDA, including single markers [31] multiple biomarker panels [32], and immune-based proteomic panels [33]. For example, a recent study of oral flora showed that specific phylotypes were associated with increased risk for developing PDA [34]. Other recent work identified new-onset diabetes as a biomarker for early pancreatic cancer [35, 36].

*K-Ras* is mutated or altered in 95% of all PDA cases; p16 is mutated or altered in 95%; p53 is mutated or altered in 75%; Smad4 is mutated or altered in 55% [37]. Mutations of any of these genes are associated with a poor PDA prognosis [3]. Regarding PLS3, one recent study that included 207 PDA tissue specimens showed that the overexpression of PLS3 was associated with tumor stage and pathology as well as poor OS in PDA patients. PLS3 was an independent prognostic factor in PDA patients [14]. The authors’ in vitro data demonstrated that PLS3 expression induced PDA cell proliferation and invasion, which were associated with increased PI3K/AKT activity in PDA cells [14]. Our current data support these recent reports of the role of PLS3 in PDA [14]. Nevertheless, there have been a number of studies of PLS3 in other human cancers, including gastric, colorectal, and breast cancers, as well as cutaneous T-cell lymphoma and acute myeloid leukemia [13–19].

PPL is an extremely rare form of malignant lymphoma with histology characteristic of DLBCL. Similarly to stage-matched DLBCL in the other organs, the condition can be treated with CHOP chemotherapy, which achieves equivalent outcomes [8]. One previous case report described the challenges in diagnosing DLBCL with an intra-sinusoidal pseudoglandular growth that mimicked poorly differentiated metastatic PDA in an intra-abdominal lymph node [38]. It is difficult to differentiate these DLBCLs from PDA [39, 40]. However, our current study did not include a direct comparison of the microarray data for PDA and the other three tissue types. Instead, we performed indirect analyses of differences in gene expression profiles between PDA and each one of the three other types. We believe that such analyses could be informative and the conclusion was validated by qPCR (Fig. 10). Our current study provided data on the differential diagnosis for PDA and DLBCL. We identified DEGs that were expressed at significantly higher levels in pancreas, compared with lymph nodes and DLBCL, including PLS3. PLS3 expression was also confirmed to significantly differ between PDA and the pancreas, which was consistent with a recent study of PLS3 in PDA [14]. Moreover, we applied bioinformatic methods to highlight the potential role of PLS3 as an improved PDA marker with differentially diagnostic value for DLBCL that a traditional tumor marker may not have.

We further verified the association of PLS3 expression with other PDA biomarkers, like KRT7 and KRT19 [41], and SPARC and SPP1. For example, SPARC was reported to affect tumor cell proliferation and migration by activating PI3K/AKT signaling and the epithelial–mesenchymal transition in liver, lung, and head and neck cancers [42–44]. SPP1 was associated with the development of gastric cancer [45]. However, their interaction in PDA needs to be further investigated.

Overall, our current study has several advantages. We used the best of our knowledge to assess PLS3 as a biomarker to aid in the differential diagnosis of PDA from DLBCL. We endeavored to collect gene expression data from multiple databases, ultimately obtaining a total of 681 PDA, 361 normal pancreas, 69 DLBCL, and 14 lymph node samples, leading to a large sample size. Measurements of PLS3 are feasible because PLS3 levels can be detected in circulating tumor cells, indicating the potential value of PLS3 as a serum tumor marker [17, 46, 47]. However, our current study does have some limitations. For example, our current data did not allow for conclusions as to whether PLS3 overexpression occurred consistently in all PDA cells throughout our clinically heterogeneous population. Moreover, low levels of PLS3 expression may prevent accurate PPL diagnosis because of the inability to rule out the possibility of secondary pancreatic lymphoma. In addition, our current study did not include other types of pancreatic cancers, like neuroendocrine tumors, follicular lymphoma, and Hodgkin’s lymphoma. Again, technically, our filter criteria for DEGs were extremely stringent, resulting in the inevitable loss of some useful information. Our current study utilized data from different online databases and datasets, leading to different numbers of cases and comparison groups, which sometimes made comparisons difficult. For example, we were only able to associate 16 DEGs (not 46 or 84) with patient survival (because these 16 DEGs were verified in the MERAV, GEO, and TCGA databases).

## Conclusions

Our current study demonstrated that the detection of PLS3 expression may be useful in diagnosing PDA, predicting PDA prognosis, and distinguishing PDA from DLBCL. Further study will be needed to verify our current data on PLS3 as a biomarker or even as a novel target for PDA therapy.

## Supplementary Information

**Additional file 1: Table S1.** Clinicopathological data of the patients involved in Cox regression analysis, Kaplan-Meier analysis and RT-qPCR analysis. The data of TCGA, R2 and ICGC was downloaded from the web.

**Additional file 2: Figure S1.** Association of tumor N classification with PDA prognosis using TCGA database. TCGA data were downloaded from the web and statistically analyzed using the Kaplan–Meier curves and the log-rank test.

## Data Availability

The datasets generated and analyzed during the present study are available from the corresponding author on reasonable request.

## Abbreviations

AUC: Area under the curve
DEG: Differentially expressed gene
DLBCL: Diffuse large B-cell lymphoma
EMT: Epithelial–mesenchymal transition
EUS-FNA: Endoscopic ultrasound-guided fine-needle aspiration biopsy
GEO: Gene Expression Omnibus
GO: Gene Ontology
GTEx: The Genotype-Tissue Expression
ICGC: International Cancer Genome Consortium
KCNK1: Potassium channel, two pore domain subfamily K, member 1
KRT19: Keratin 19
KRT7: Keratin 7
MAL2: Mal, T-cell differentiation protein 2
MERAV: Metabolic gEne RApid Visualizer
NET1: Neuroepithelial cell transforming 1
OS: Overall survival
PDA: Pancreatic ductal adenocarcinoma
PLS1: Plastin-1
PLS3: Plastin-3
PPL: Primary pancreatic lymphoma
ROC: Receiver operating characteristic curve
r_s_: Spearman’s rank correlation coefficient
SPARC: Secreted protein acidic and cysteine rich
SPP1: Secreted phosphoprotein 1
TCGA: The Cancer Genome Atlas

## References

[1] Wild C, Weiderpass E, Stewart BW (2020). Pancreatic cancer, world cancer report: cancer research for cancer prevention.

[2] Rahib L, Smith BD, Aizenberg R, Rosenzweig AB, Fleshman JM, Matrisian LM (2014). Projecting cancer incidence and deaths to 2030: the unexpected burden of thyroid, liver, and pancreas cancers in the United States. Cancer Res.

[3] Wolfgang CL, Herman JM, Laheru DA, Klein AP, Erdek MA, Fishman EK (2013). Recent progress in pancreatic cancer. CA Cancer J Clin.

[4] Vincent A, Herman J, Schulick R, Hruban RH, Goggins M (2011). Pancreatic cancer. Lancet.

[5] Kamisawa T, Wood LD, Itoi T, Takaori K (2016). Pancreatic cancer. Lancet.

[6] Urrutia G, Salmonson A, Toro-Zapata J, de Assuncao TM, Mathison A, Dusetti N (2020). Combined targeting of g9a and checkpoint kinase 1 synergistically inhibits pancreatic cancer cell growth by replication fork collapse. Mol Cancer Res.

[7] Mukhija D, Nagpal SJS, Sohal DPS (2019). Epidemiology, tumor characteristics, and survival in patients with primary pancreatic lymphoma: a large population-based study using the seer database. Am J Clin Oncol.

[8] Zheng SM, Zhou DJ, Chen YH, Jiang R, Wang YX, Zhang Y (2017). Pancreatic T/histiocyte-rich large B-cell lymphoma: a case report and review of literature. World J Gastroenterol.

[9] Sadot E, Yahalom J, Do RK, Teruya-Feldstein J, Allen PJ, Gonen M (2015). Clinical features and outcome of primary pancreatic lymphoma. Ann Surg Oncol.

[10] Khashab M, Mokadem M, DeWitt J, Emerson R, Sherman S, LeBlanc J (2010). Endoscopic ultrasound-guided fine-needle aspiration with or without flow cytometry for the diagnosis of primary pancreatic lymphoma—a case series. Endoscopy.

[11] Savari O, Al-Duwal Z, Wang Z, Ganesan S, Danan-Rayes R, Ayub S (2020). Pancreatic lymphoma: a cytologic diagnosis challenge. Diagn Cytopathol.

[12] Huang Z, Li M, He D, Wei Y, Yu H, Wang Y (2019). Two-dimensional texture analysis based on CT images to differentiate pancreatic lymphoma and pancreatic adenocarcinoma: a preliminary study. Acad Radiol.

[13] Kurashige J, Yokobori T, Mima K, Sawada G, Takahashi Y, Ueo H (2019). Plastin3 is associated with epithelial–mesenchymal transition and poor prognosis in gastric cancer. Oncol Lett.

[14] Xin Z, Li D, Mao F, Du Y, Wang X, Xu P (2020). PLS3 predicts poor prognosis in pancreatic cancer and promotes cancer cell proliferation via PI3K/AKT signaling. J Cell Physiol.

[15] Kujawski R, Przybyłowska-Sygut K, Mik M, Lewandowski M, Trzciński R, Berut M (2015). Expression of the pls3 gene in circulating cells in patients with colorectal cancer. Pol Przegl Chir.

[16] Velthaus A, Cornils K, Hennigs JK, Grub S, Stamm H, Wicklein D (2019). The actin binding protein plastin-3 is involved in the pathogenesis of acute myeloid leukemia. Cancers.

[17] Ueo H, Sugimachi K, Gorges TM, Bartkowiak K, Yokobori T, Muller V (2015). Circulating tumour cell-derived plastin3 is a novel marker for predicting long-term prognosis in patients with breast cancer. Br J Cancer.

[18] Yokobori T, Iinuma H, Shimamura T, Imoto S, Sugimachi K, Ishii H (2013). Plastin3 is a novel marker for circulating tumor cells undergoing the epithelial–mesenchymal transition and is associated with colorectal cancer prognosis. Cancer Res.

[19] Szkandera J, Winder T, Stotz M, Weissmueller M, Langsenlehner T, Pichler M (2013). A common gene variant in PLS3 predicts colon cancer recurrence in women. Tumour Biol.

[20] Shaul YD, Yuan B, Thiru P, Nutter-Upham A, McCallum S, Lanzkron C (2016). MERAV: a tool for comparing gene expression across human tissues and cell types. Nucleic Acids Res.

[21] Ritchie ME, Phipson B, Wu D, Hu Y, Law CW, Shi W (2015). limma powers differential expression analyses for RNA-sequencing and microarray studies. Nucleic Acids Res.

[22] Leek JT, Johnson WE, Parker HS, Jaffe AE, Storey JD (2012). The sva package for removing batch effects and other unwanted variation in high-throughput experiments. Bioinformatics.

[23] Yu G, Wang LG, Han Y, He QY (2012). clusterProfiler: an R package for comparing biological themes among gene clusters. OMICS.

[24] Walter W, Sanchez-Cabo F, Ricote M (2015). GOplot: an R package for visually combining expression data with functional analysis. Bioinformatics.

[25] Bailey P, Chang DK, Nones K, Johns AL, Patch AM, Gingras MC (2016). Genomic analyses identify molecular subtypes of pancreatic cancer. Nature.

[26] Robin X, Turck N, Hainard A, Tiberti N, Lisacek F, Sanchez JC (2011). pROC: an open-source package for R and S+ to analyze and compare ROC curves. BMC Bioinform.

[27] Hornick JL, Lauwers GY, Odze RD (2005). Immunohistochemistry can help distinguish metastatic pancreatic adenocarcinomas from bile duct adenomas and hamartomas of the liver. Am J Surg Pathol.

[28] Huo Z, Zhai S, Weng Y, Qian H, Tang X, Shi Y (2019). PRPF40A as a potential diagnostic and prognostic marker is upregulated in pancreatic cancer tissues and cell lines: an integrated bioinformatics data analysis. Onco Targets Ther.

[29] Ma YY, Shi JJ, Chen JB, Xu KC, Niu LZ (2020). Irreversible electroporation for liver metastasis from pancreatic cancer: a case report. World J Clin Cases.

[30] Fritscher-Ravens A (2003). Endoscopic ultrasound evaluation in the diagnosis and staging of lung cancer. Lung Cancer.

[31] Kim J, Bamlet WR, Oberg AL, Chaffee KG, Donahue G, Cao XJ (2017). Detection of early pancreatic ductal adenocarcinoma with thrombospondin-2 and CA19–9 blood markers. Sci Transl Med.

[32] Cohen JD, Javed AA, Thoburn C, Wong F, Tie J, Gibbs P (2017). Combined circulating tumor DNA and protein biomarker-based liquid biopsy for the earlier detection of pancreatic cancers. Proc Natl Acad Sci USA.

[33] Mellby LD, Nyberg AP, Johansen JS, Wingren C, Nordestgaard BG, Bojesen SE (2018). Serum biomarker signature-based liquid biopsy for diagnosis of early-stage pancreatic cancer. J Clin Oncol.

[34] Fan X, Alekseyenko AV, Wu J, Peters BA, Jacobs EJ, Gapstur SM (2018). Human oral microbiome and prospective risk for pancreatic cancer: a population-based nested case–control study. Gut.

[35] Sharma A, Smyrk TC, Levy MJ, Topazian MA, Chari ST (2018). Fasting blood glucose levels provide estimate of duration and progression of pancreatic cancer before diagnosis. Gastroenterology.

[36] Sharma A, Kandlakunta H, Nagpal SJS, Feng Z, Hoos W, Petersen GM (2018). Model to determine risk of pancreatic cancer in patients with new-onset diabetes. Gastroenterology.

[37] Ryan DP, Hong TS, Bardeesy N (2014). Pancreatic adenocarcinoma. New Engl J Med.

[38] Yau D, Aron M, Siddiqi IN (2019). Diffuse large B-cell lymphoma with striking intrasinusoidal pseudoglandular growth pattern as a diagnostic dilemma mimicking metastatic poorly differentiated pancreatic adenocarcinoma in an intraabdominal lymph node. Int J Surg Pathol.

[39] Constantin A, Tănase AD, Săftoiu A, Copăescu C (2018). A primary retroperitoneal diffuse large b-cell lymphoma: a challenging diagnosis. Curr Health Sci J.

[40] Ravindhran B, Prakash C, Govindharaj S, Bahnou NMS, Pavithra B (2017). An aggressive primary retroperitoneal diffuse large B-cell lymphoma mimicking a pancreatic neoplasm, presenting as duodenal stenosis. J Clin Diagn Res.

[41] Neureiter D, Zopf S, Dimmler A, Stintzing S, Hahn EG, Kirchner T (2005). Different capabilities of morphological pattern formation and its association with the expression of differentiation markers in a xenograft model of human pancreatic cancer cell lines. Pancreatology.

[42] Chang CH, Yen MC, Liao SH, Hsu YL, Lai CS, Chang KP (2017). Secreted protein acidic and rich in cysteine (SPARC) enhances cell proliferation, migration, and epithelial mesenchymal transition, and SPARC expression is associated with tumor grade in head and neck cancer. Int J Mol Sci.

[43] Wan S, Meyer AS, Weiler SME, Rupp C, Toth M, Sticht C (2018). Cytoplasmic localization of the cell polarity factor scribble supports liver tumor formation and tumor cell invasiveness. Hepatology.

[44] Sun W, Feng J, Yi Q, Xu X, Chen Y, Tang L (2018). SPARC acts as a mediator of TGF-beta1 in promoting epithelial-to-mesenchymal transition in A549 and H1299 lung cancer cells. BioFactors.

[45] Song SZ, Lin S, Liu JN, Zhang MB, Du YT, Zhang DD (2019). Targeting of SPP1 by microRNA-340 inhibits gastric cancer cell epithelial–mesenchymal transition through inhibition of the PI3K/AKT signaling pathway. J Cell Physiol.

[46] Shi F, Ma Y, Qian Y, Wang Y, Wang Z, Zhao M (2019). A novel peptide probe for identification of PLS3-expressed cancer cells. Anal Chem.

[47] Markiewicz A, Topa J, Nagel A, Skokowski J, Seroczynska B, Stokowy T (2019). Spectrum of epithelial–mesenchymal transition phenotypes in circulating tumour cells from early breast cancer patients. Cancers.

