# Supplementary figures and images for: Plastin-3 is a diagnostic and prognostic marker for pancreatic adenocarcinoma and distinguishes from diffuse large B-cell lymphoma
Source: Cancer Cell Int. 2021 Aug 4;21:411. doi: 10.1186/s12935-021-02117-1 (PMC8336331; doi:10.1186/s12935-021-02117-1)

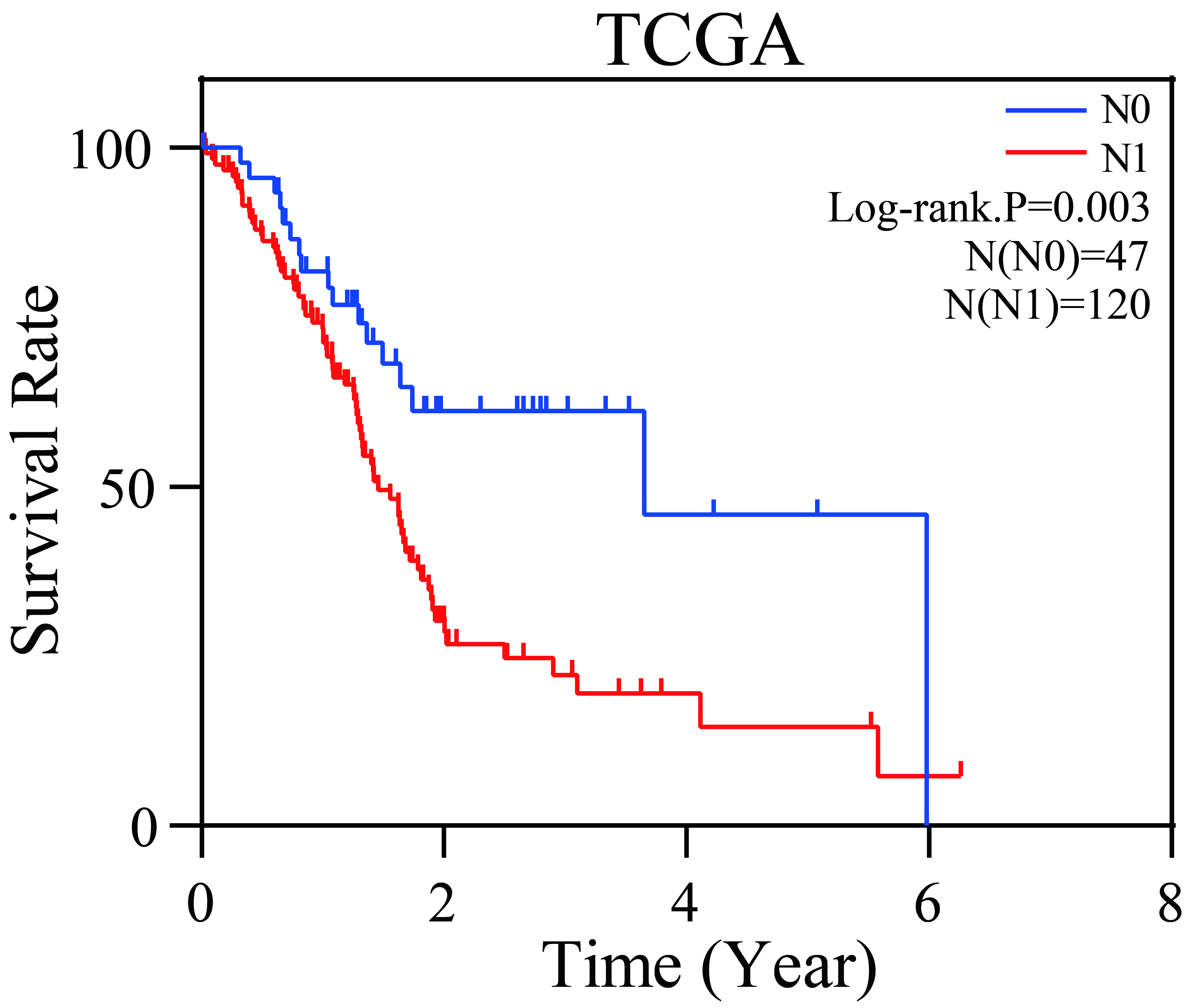

Supplement: Supplementary file 2 — Additional file 2: Figure S1. Association of tumor N classification with PDA prognosis using TCGA database. TCGA data were downloaded from the web and statistically analyzed using the Kaplan–Meier curves and the log-rank test. [file 12935_2021_2117_MOESM2_ESM.tif]
